# A familial missense ACTA2 variant p.Arg198Cys leading to Moyamoya-like arteriopathy with straight course of the intracranial arteries, aortic aneurysm and lethal aortic dissection

**DOI:** 10.1186/s42466-023-00268-2

**Published:** 2023-08-17

**Authors:** Jan K. Focke, Markus Kraemer

**Affiliations:** 1 Department of Neurology, Alfried Krupp Hospital, Essen, Germany; 2 Department of Neurology, Heinrich-Heine-University Hospital, Düsseldorf, Germany

**Keywords:** *ACTA2* variant, Cerebral vasculopathies, Angiography, Moyamoya angiopathy, Aortic dissections

## Abstract

**Background:**

Cerebral vasculopathies frequently lead to severe medical conditions such as stroke or intracranial hemorrhage and have a broad range of possible etiologies that require different therapeutic regimens. However, vasculopathies sometimes present with characteristic angiographic findings, that — if recognized — can guide a more specific diagnostic work-up. Certain *ACTA2* variants are associated with a distinctive cerebrovascular phenotype characterized by an anomalously straight course of intracranial arteries, dilatation of proximal ICA and stenosis of distal ICA, in the absence of a compensatory basal collateral network found in Moyamoya disease. Until recently, this *ACTA2* cerebral arteriopathy has been reported only in *ACTA2* variants impairing Arg179.

**Methods and materials:**

We report a familial case of a missense *ACTA2* variant p.Arg198Cys with angiographic features of an *ACTA2* cerebral arteriopathy. We analyzed the neuroimaging features of all four variant carrying family members and discussed the cerebrovascular abnormalities we found on the background of the current literature on *ACTA2* arteriopathies.

**Results:**

Neuroimaging of the variant carriers revealed angiographic abnormalities characteristic for *ACTA2* cerebral arteriopathy such as stenoses of the terminal internal carotid artery, occlusion of the proximal middle cerebral artery and an anomalously straight course of the intracranial arteries. In our index patient catheter angiography showed a Moyamoya-like basal collateral network alongside with the above-mentioned features of an *ACTA2* cerebral arteriopathy. The detected missense *ACTA2* variant p.Arg198Cys was not known to be associated a cerebral arteriopathy, so far. One of the patients later died from aortic dissection — a common vascular complication of *ACTA2* variants.

**Conclusion:**

The familial case expands the phenotype of the detected *ACTA2* variant p.Arg198Cys and hereby broadens the range of *ACTA2* variants associated with a cerebral arteriopathy. Further, it emphasizes the importance of an interdisciplinary approach of vasculopathies.

## Background

Smooth muscle cell alpha-actin, encoded by *ACTA2*, is a key component of the contractile apparatus in smooth muscle cells of blood vessels and hollow organs [1]. Accordingly, heterozygous pathogenic variants of *ACTA2* are associated with a wide spectrum of vascular but also systemic disorders. Thoracic aortic aneurysms and dissections (TAAD), occlusive vascular diseases such as juvenile strokes and coronary artery disease (CAD) are the most common and clinically relevant vascular manifestations of *ACTA2* variants [2]. *ACTA2* variants impairing Arg179 are associated with a particularly severe but also distinctive multisystemic phenotype summarized as syndrome of multisystemic smooth muscle dysfunction (SMDS) that exhibits congenital mydriasis, intestinal hypoperistalsis, hypotonic bladder, pulmonary hypertension, aortic disease and Moyamoya-like cerebral arteriopathy [2–4].

While in the past cerebrovascular abnormalities associated with *ACTA2* variants were regarded as a variant of Moyamoya angiopathy (MMA), in recent years — due to its unique radiologic and histopathologic features — *ACTA2* cerebral arteriopathy has been established as a novel entity independent of MMA [5]. Radiologic features of *ACTA2* cerebral arteriopathy are a straight appearance of the intracranial arteries, dilatation of the proximal internal carotid artery (ICA) and stenosis of the distal ICA, in absence of the compensatory basal collateral network characteristic for Moyamoya disease [3–5]. Histopathologically, fibromuscular proliferation of the intimal and medial layer of cerebral arterial walls resulting in increased wall thickness and narrowing of the lumen might account for occlusive vascular complications as well as for the pathognomonic straight appearance of intracranial arteries in *ACAT2* patients [1]. Fragmentation and loss of the elastic lamellae of the aorta might be causal for the increased risk of aortic aneurysms and dissection due to decreased resistance to blood pressure [1]. This distinctive cerebrovascular phenotype has first been described in patients with SMDS resulting from *ACTA2* variants impairing Arg179 [4]. However, recently Kaw et al. published five cases of patients with *ACTA2* variants and phenotypic features of SMDS, among whom four cases presenting variants not involving Arg179 [6]. This indicates that a broader range of *ACTA2* variants manifest with features of SMDS. Consistent with this notion, features of *ACTA2* cerebral arteriopathy have been observed in several variants that do not involve Arg179 (p.Arg258Cys, p.Arg258His, p.Asn117Lys, p.Asp181Val, p.Met46Arg) [6–9].

## Methods and material

The clinical manifestations, medical history, laboratory and neuroimaging findings of four related carriers of the missense *ACTA2* variant c.592C > T p.Arg198Cys were analyzed. Neuroimaging of all variant carrying family members was reviewed on the background of current literature on *ACTA2* cerebral arteriopathy. For genetic testing DNA was isolated from whole blood samples of the patients. Based on the angiographic indication of an *ACTA2* cerebral arteriopathy a targeted sequencing of the *ACTA2* exons was performed.

## Results/case description

**Fig. 1:**
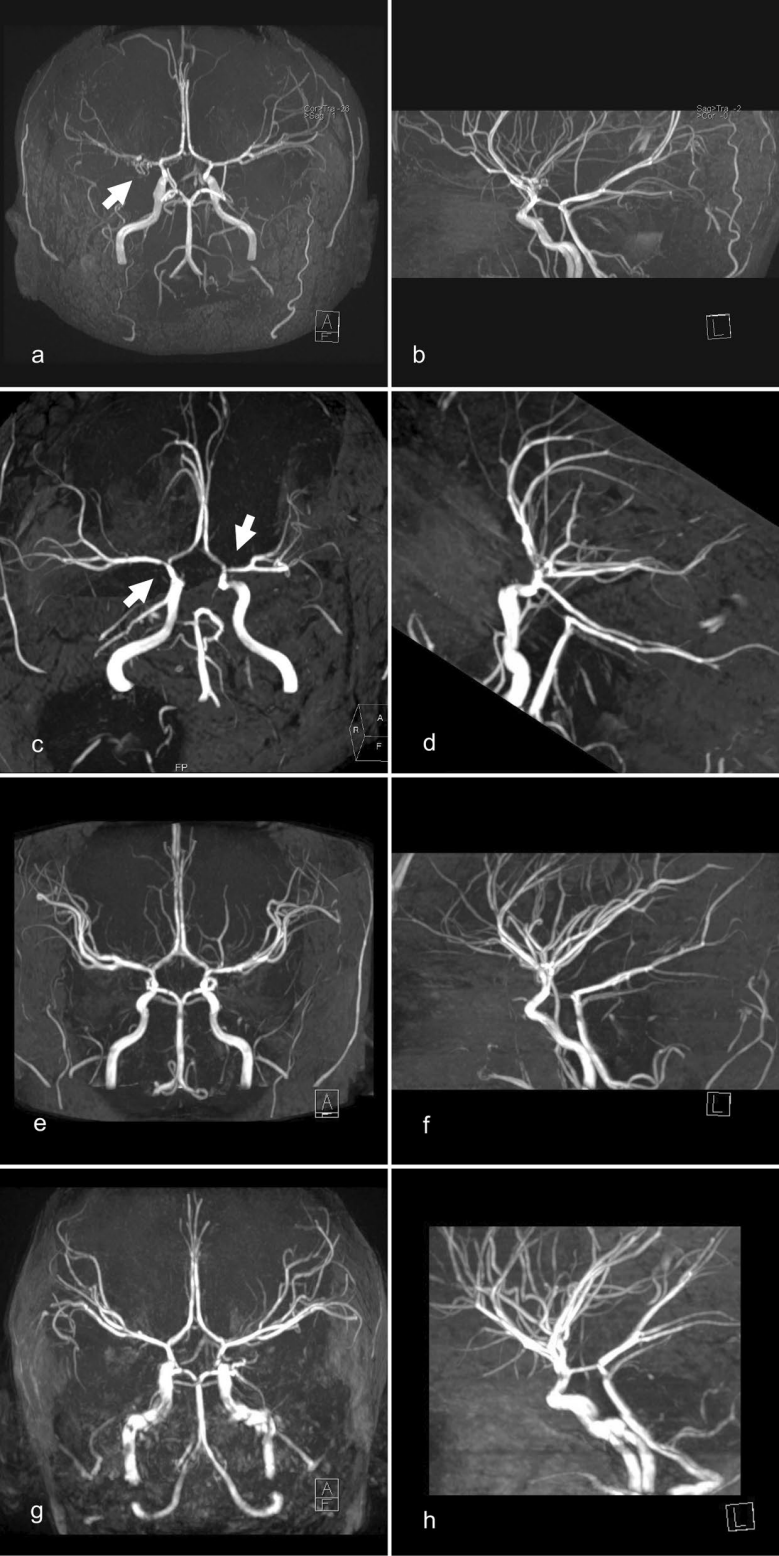
**a** Planar and **b** lateral view of the intracranial magnet resonance angiography (MRA) of the index patient showing Moyamoya-like features of the right middle cerebral artery (MCA) (arrow) as well as an anomalously straight course of the cerebral arteries; **c** planar and **d** lateral view of the intracranial MRA of the index patient’s mother also exhibiting straight cerebral arteries as well as bilateral stenosis of the terminal internal carotid artery (ICA) and proximal MCA (arrows); **e** planar and **f** lateral view of the intracranial MRA of the index patient’s daughter and son (**g**; **h**) with straight cerebral arteries

**Fig. 2:**
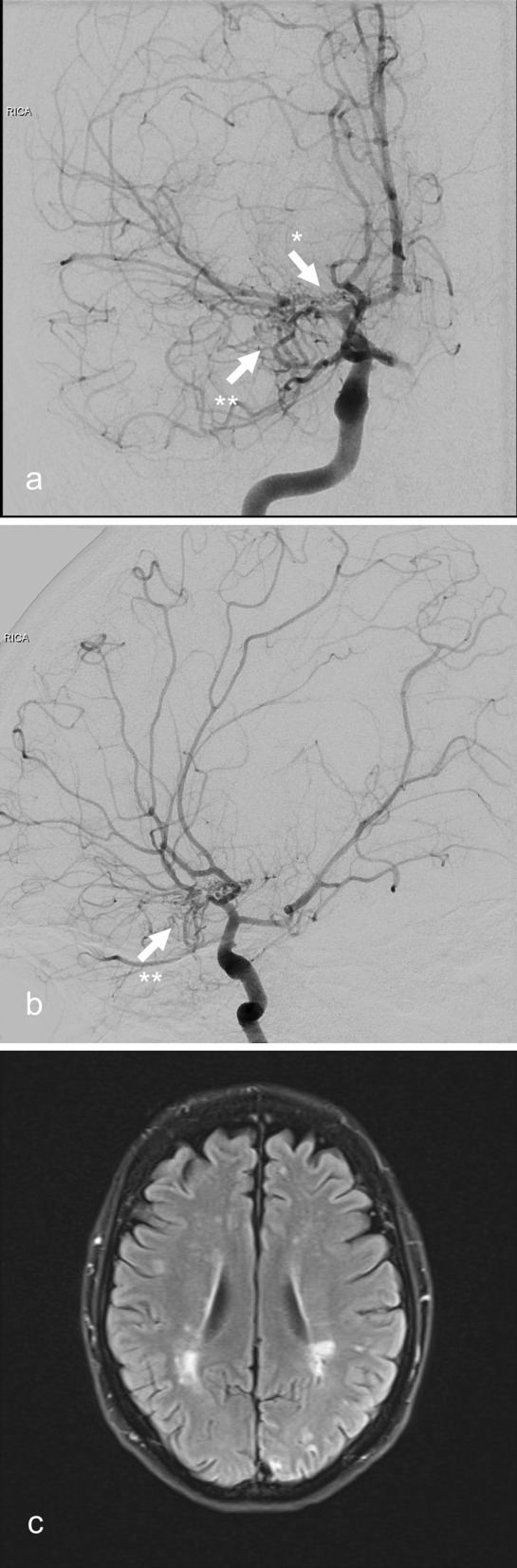
**a** Frontal and **b** lateral view of the catheter angiography of the index patient showing stenoses of the terminal right ICA and occlusion of the proximal right MCA (arrow with asterisk) as well as a Moyamoya-like basal collateral network (arrow with double asterisk); **c** MRI of the index patient’s mother showing white matter hyperintensities

In 2020, a 40-years-old woman was referred to our department of neurology for further diagnostic evaluation of the tentative diagnosis of MMA. The referral took place after the patient had developed recurring episodes of severe headache after physical activity and magnetic resonance imaging (MRI) had shown features of a unilateral variant of MMA with stenoses of the terminal right ICA, occlusion of the proximal right middle cerebral artery (MCA) and a basal collateral network, in the absence of white matter hyperintensities. Preexisting medical conditions were a malignant melanoma and a lumbar ependymoma — both successfully resected — as well as an umbilical hernia. Family history included amyotrophic lateral sclerosis in the patient’s father as well as the decease of her maternal grandmother at the age of 35 years due to unknown reason. At the time of proband’s assessment no family history of TAAD was recorded. Clinical examination revealed no neurologic deficits. Laboratory investigations including cerebrospinal fluid (CSF) analysis yielded no abnormalities of clinical relevance. The perfusion MRI revealed a cerebral perfusion deficit corresponding with the right sided MCA stenosis. In addition to the above-mentioned Moyamoya-like vascular features magnetic resonance and catheter angiography at our facility showed an anomalously straight course of the cerebral arteries suggestive of *ACTA2* variants as well as a basal collateral network (Figs. 1a, b and 2a, b). Subsequently, genetic testing revealed a heterozygous *ACTA2* variant c.592C > T p.Arg198Cys. The detected variant is listed in population databases with a minor allele frequency of 0.00001 (gnomAD). It has been submitted to ClinVar database four times in distinct subjects with TAAD. In the literature, this ACTA2 variant has previously been reported in two unrelated cases of early-onset TAAD, with occurrence of aortic events at 12 and 32 years of age [10]. In silico prediction tools (PolyPhen, SIFT) have indicated that the p.Arg198Cys variant disrupts protein function and classified it as probably deleterious. Based on its above-mentioned rarity in controls, recurrence in association with TAAD and in silico prediction of deleterious effects the p.Arg198Cys *ACTA2* variant was classified as likely pathogenic in accordance with the American College of Medical Genetics and Genomics (ACMG) guidelines [11].

**Fig. 3:**
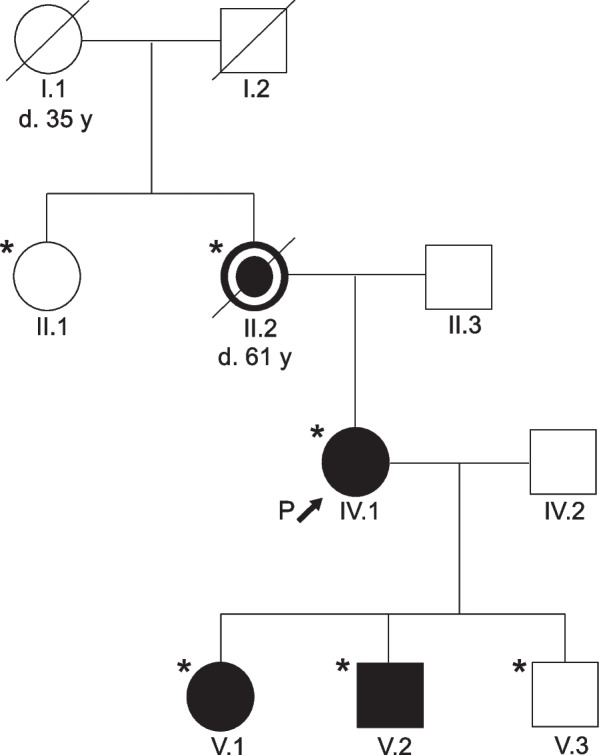
Pedigree of the family. The index patient is indicated by an arrow. Empty symbols designate healthy individuals and full symbols designate family members affected by the p.(Arg198Cys) *ACTA2* variant. Among the affected indivuals blackened symbols designate individuals with *ACTA2* cerebral arteriopathy and blackened symbols with a white circle designate individuals with both *ACTA2* cerebral arteriopathy and aortic dissection. Asterisks indicate individuals that underwent genetic screening

Due to the increased risk of aortic aneurysms and dissections associated with *ACTA2* variants an MRI of the aorta was performed that ruled out aortic ectasia or aneurysm in our patient. Transesophageal echocardiography revealed the absence of a patent ductus arteriosus and ophthalmologic examination revealed absence of mydriasis as further abnormalities associated with *ACTA2* variants. To minimize the risk of stroke indicated by impaired perfusion of the right MCA territory an extra- to intracranial bypass between the superficial temporal artery and MCA was operated and an antiplatelet therapy with 100 mg acetylsalicylic acid per day was initiated.

Genetic testing of the patient’s relatives revealed the same *ACTA2* variant carried by the patient’s mother (61 years old; Fig. 3 II.2), her daughter (12 years old; Fig. 3 V.1) and one of her two sons (9 years old; Fig. 3 V.2). The fraternal twin brother of the affected son as well as the index patient’s maternal aunt were not found to be variant carriers. All mutated relatives were asymptomatic at the time of the genetic screening. The cranial MRI of the patient’s mother (Fig. 3 II.2) showed white matter hyperintensities (Fig. 2c) as well as features of *ACTA2* cerebral arteriopathy such as the characteristic abnormally straight course of intracranial arteries, bilateral stenosis of the terminal ICA, proximal MCA and anterior cerebral artery (ACA) (Fig. 1c, d). Thoracic computed tomography (CT) revealed no aortic ectasia or aneurysm. However, the index patient’s mother suffered a dissection of the descending thoracic aorta in 2021 that was successfully treated by endovascular stenting. Later that year she died from aortic rupture. The cranial MRI of the index patient’s variant carrying children (Fig. 3 V.1., V.2) also showed the characteristic straight course of intracranial arteries (Fig. 1e–h). The children’s transthoracic echocardiography revealed the absence of a patent ductus arteriosus.

## Discussion

Despite intensive diagnostic work-up the etiology of around 30% of strokes remains unknown [12]. Although rare, genetic disorders as *ACTA2* variants are important to consider especially in juvenile and familial stroke cases which are not sufficiently explained by conventional risk factors. The described familial case expands the phenotype of the *ACTA2* variant c.592C > T p.Arg198Cys, which formerly has been reported only in connection with TAAD in two individuals [10]. Our case is the first demonstrating the association of this missense variant with features of Moyamoya-like *ACTA2* cerebral arteriopathy. *ACTA2* cerebral arteriopathy is characterized by the pathognomonic straight course of intracranial arteries, dilatation of proximal ICA and stenosis of distal ICA, in the absence of a basal Moyamoya collateral network [3–5]. This distinctive cerebrovascular phenotype has been described first in patients with SMDS resulting from *ACTA2* variants impairing Arg179 [4]. Recently, growing evidence has emerged suggesting that certain features of SMDS including *ACTA2* cerebral arteriopathy do not exclusively occur in variants involving Arg179 but are present in a broader range of *ACTA2* variants [6–9]. In the described case all four variant carrying family members exhibited features of *ACTA2* cerebral arteriopathy. The particularly characteristic straight course of the intracranial arteries was present in all four variant carriers (Fig. 1a–h). The reported familial case hereby expands the genotype of *ACTA2* cerebral arteriopathy. Notably, in our index patient alongside with characteristic features of an *ACTA2* cerebral arteriopathy a Moyamoya-like basal collateral network was present, although not as highly developed as typically encountered in MMA (Figs. 1a, 2a, b) [13]. A dilatation of the proximal ICA — a repeatedly described feature of *ACTA2* cerebral arteriopathy — was not present in any of the reported patients. This suggests a certain phenotypic heterogeneity of *ACTA2* cerebral arteriopathy that might be attributed to the respective variant. However, an irregularly straight course of intracranial arteries is a highly recognizable radiologic feature that should prompt genetic testing for *ACTA2* variants, even more so in patients who present several features of SMDS or a personal or family history of TAAD. However, the reported familial case demonstrates that genetic screening for *ACTA2* variants should also be considered in Moyamoya-like arteriopathy with a straight course of intracranial arteries, even in the absence of a family history of TAAD or features of SMDS, and even if a basal collateral network is found. Identifying *ACTA2* arteriopathy in patients suffering from neurological disorders and differentiating it from other cerebrovascular conditions such as cerebral atherosclerosis, vasculitis or MMA is of high relevance for the early detection of patients at risk of aortic dissection [5]. According to Milewicz and Regalado, early diagnosis of the variant and subsequent aortic surveillance, blood pressure control, and timely surgical repair of ascending aortic aneurysms, may help to reduce the risk of aortic complications [14, 15]. Notably, in *ACTA2* variants, one third of the patients dissected at aortic diameters less than the diameter recommended for surgical repair of 5.0 cm. Therefore, surgical repair of aortic dilatations should be considered already at a diameter of 4.5 cm [14].

The reported case shows that the recognition of characteristic cerebrovascular abnormalities such as a straight course of intracranial arteries can guide the detection of an *ACTA2* variant and this in turn can lead to the detection of a thoracic aortic aneurysm. Thus knowledge of the underlying genetic mutation can guide personalized aortic surveillance and hereby prevent aortic dissection and death. This case emphasizes the importance of an interdisciplinary approach of vasculopathies as the focus on only one organ system bears the danger of overlooking severe complications in others.

## Conclusion

To the best of our knowledge this is the first time features of an *ACTA2* cerebral arteriopathy have been found in patients carrying the p.Arg198Cys *ACTA2* variant. Interestingly, a Moyamoya-like collateral network — usually absent in the context of *ACTA2* variants — was found in our index patient, whereas a dilation of the proximal ICA — repeatedly described in other cases of *ACTA2* cerebral arteriopathy — was not present in any of our patients. Although some variability can be observed in the cerebrovascular phenotype of distinct *ACTA2* variants the anomalously straight course of the intracranial arteries is consistent and highly recognizable for *ACTA2* cerebral arteriopathy. Another novelty of the reported familial case is the isolated nature of the Moyamoya-like arteriopathy in three out of four of our patients. Aside from the index patient’s mother who suffered an aortic dissection, no *ACTA2* characteristics other than a Moyamoya-like arteriopathy were encountered in our patients, whereas *ACTA2* mutated individuals with a Moyamoya-like arteriopathy reported in the literature typically present other *ACTA2* features. Despite its rarity *ACTA2* variants are important to diagnose as early detection can help to prevent severe vascular complications as aortic dissection, coronary artery occlusion and ischemic stroke.

## Data Availability

Not applicable as no datasets were generated or analysed.

## Abbreviations

*ACTA2*: Alpha actin 2
*ACA*: Anterior cerebral artery
*CAD*: Coronary artery occlusion
*CSF*: Cerebrospinal fluid
*CT*: Computed tomography
*ICA*: Internal carotid artery
*MCA*: Middle cerebral artery
*MMA*: Moyamoya angiopathy
*MRA*: Magnetic resonance imaging
*MRI*: Magnetic resonance angiography
*SMDS*: Multisystemic smooth muscle dysfunction
*TAAD*: Thoracic aortic aneurysms and dissections
